# Advances in Musculoskeletal Cell Therapy: Basic Science and Translational Approaches

**DOI:** 10.3390/cells11233858

**Published:** 2022-11-30

**Authors:** Enrico Ragni, Alessandra Colombini, Silvia Lopa

**Affiliations:** 1 Laboratorio di Biotecnologie Applicate all’Ortopedia, IRCCS Istituto Ortopedico Galeazzi, Via R. Galeazzi 4, I-20161 Milan, Italy; 2 Cell and Tissue Engineering Laboratory, IRCCS Istituto Ortopedico Galeazzi, Via R. Galeazzi 4, I-20161 Milan, Italy

Nowadays, the real need in orthopedic research is to strictly validate advanced regenerative medicine approaches in preclinical models, with the hope that this unique and straightforward approach can facilitate a safe and effective translation into everyday clinical practice. To this aim, a profound comprehension of progenitor cells, stem- or tissue-specific, as well as their activity in pathological tissues and patients, is mandatory, thus making cell-based and cell-free therapies the new frontier for the most advanced musculoskeletal treatments.

This Special Issue aims to provide an overview of the techniques and results framing cutting-edge cell therapies for pathologies of the musculoskeletal system, from basic science to clinical studies.

Considering the competent nature of cells, which enables them to respond to external stimuli, culturing conditions can deeply affect their therapeutic or healing potential. Nguyen et al. [1] reported the interaction between bone-marrow-derived mesenchymal stem cells (MSCs) and endothelial cells in normoxia and hypoxia. The authors clearly showed that oxygen supply strongly alters cell proliferation and differentiation. In particular, cocultured cells in normoxia retain both osteogenic differentiation and endothelial markers, while hypoxic conditions limit cell proliferation and osteogenesis in favor of the angiogenic function. The impact of culturing conditions on cell phenotypes was also investigated by Carluccio et al. [2], who used platelet lysate (PL) as a supplement for human articular chondrocytes and cartilage explants. PL was able to induce the release of cartilage-derived cells from ex vivo cultured cartilage chips. Remarkably, these cells showed high proliferation rates and clonogenic abilities, and also formed hyaline cartilage-like tissue in vivo following ectopic subcutaneous implantation in mice. In addition, PL influenced the paracrine activity of chondroprogenitor cells, fostering the debate on the use of this specific population as a suitable candidate for therapies counteracting cell hypertrophy in articular cartilage. Besides establishing appropriate culture conditions and characterizing their influence on cell phenotypes, one important issue when producing and analyzing in vitro data involves the correct definition of parameters which cell and molecular approaches are based on. In this context, especially for gene expression studies, the identification of reliable reference genes (RGs) is mandatory. This need was clearly described in the work presented by Ragni et al. [3], where the suitability of commonly used RGs was evaluated with bioinformatics tools in tendon cell samples cultured in standard and progenitor-enriching conditions, as well as under inflammatory or pro-fibrotic/healing stimulation conditions. The authors showed that the gene encoding for Actin B can be reliably used when analyzing tendon cells exposed to diverse pathological conditions, marking a full stop for future molecular in vitro studies on tendons and tissue-resident cells and progenitors.

Together with cell-based approaches, cell-free products have garnered growing interest as new options in the orthopedic field. Indeed, secreted factors and extracellular vesicles (EVs), collectively named “secretome”, have shown potent regenerative and anti-inflammatory action in several contests, including orthopedic disorders. In this frame, Arrigoni et al. [4] gave a comprehensive overview of the potentiality of umbilical-cord-derived MSCs and their secretome in the therapy of arthritic diseases. Their review reports encouraging results from preclinical investigations and preliminary clinical trials applying cell-based therapies, and by pioneering works using clinical-grade EVs. These results are of fundamental importance since, compared to a cell-based product, the whole secretome or isolated EVs could have advantages in terms of the safety and ease of handling. In this frame, the authors discussed the need to develop standardized production protocols relying on the “quality by design” approach, including a series of quality and potency tests aimed at identifying the mode of action, a crucial parameter for the eventual clinical translation. Under this perspective, Ragni et al. [5] described a combination of confocal microscopy and microfluidic technologies to assess the incorporation of MSC-derived EVs in chondrocytes and synoviocytes from osteoarthritic (OA) patients. Compared to conventional 2D cultures, hydrogel-based cultures in microfluidic devices mimicking tissue organization allowed a more physiological EV–cell interaction to be recapitulated, representing a cutting-edge system to identify the molecular mechanisms underlying EV action in target cells and tissues. Once defined and validated, this strategy can become a valuable tool, not only for basic research but also for release assays and potency prediction for clinical EV batches.

Along with the characterization of molecular mechanisms or the potency of biological therapies for musculoskeletal disorders through reliable in vitro assays and models, the potential of these therapies needs to be verified in animal models before clinical translation. Promising results were obtained for tendinopathies by de Girolamo et al. [6], who showed the positive outcomes of human amniotic suspension allograft (ASA) in a rat model of Achilles tendinopathy. ASA treatment was safe and well-tolerated, and also resulted in a widespread improvement in the tendon tissue. This study provided a fundamental step regarding the use of ASA for the treatment of Achilles tendinopathy, approaching its translation into clinical practice. Nevertheless, each animal model should consider the impact of differences between recipients that may alter the model reliability, possibly over- or under-estimating the performance of the tested product. Watson et al. [7] reported an important, albeit rare, case in this perspective. In fact, authors showed that human MSCs locally introduced to femur fractures in streptozotocin-induced diabetic mice did not facilitate a more efficient bone union compared to the controls. Thus, this pilot study suggested that MSCs derived from the human bone marrow of non-diabetic donors are not able support fracture healing in diabetic mice, supporting the idea that the environment in which cells are implanted and the health conditions of the recipient can play a major role in the outcome of cell-based therapies. Under these premises, Garcia et al. [8] mapped the expression of the inflammatory cytokine oncostatin M (OSM) in the joint tissues of rats with either an acute inflammatory model or an instability-induced OA model. OSM expression correlated with cartilage damage, synovitis, and osteophyte formation. Most importantly, in vivo data highly correlated with those obtained in the synovial fluid of OA patients where detectable OSM was associated with higher levels of other inflammatory cytokines, potentially influencing the outcome of new therapies depending on the inflammatory environment.

The treatment of patients is the ultimate goal of translational research. A clear example in this direction was reported by Klimczak et al. [9] in the context of Duchenne muscular dystrophy (DMD). The authors followed the co-transplantation of two stem/progenitor cell populations, bone-marrow-derived MSCs and skeletal muscle-derived stem/progenitor cells, into the dystrophic muscle of three DMD patients. The combined administration was safe, with no reported adverse events, and was effective, as proven by the increased motor unit parameters, the decrease in creatine kinase levels, and the normalized profile of pro-inflammatory cytokines. These encouraging data form the basis for the design of larger clinical trials to optimize the timing and dosing of stem/progenitor cell delivery.

In conclusion, this Special Issue aims to demonstrate cutting-edge advancements in innovative therapies for musculoskeletal cell therapies under the polar star of a thorough and deep characterization of translational approaches from the bench to the bedside.

## Data Availability

The raw data presented in this manuscript are openly available in OSF at https://osf.io/9t78z/?view_only=b11447df2f3d463d858d941f8c39cc0e (accessed on 17 November 2022).
